# The Wnt/β-Catenin Pathway Regulates the Expression of the miR-302 Cluster in Mouse ESCs and P19 Cells

**DOI:** 10.1371/journal.pone.0075315

**Published:** 2013-09-10

**Authors:** Christien Bräutigam, Angelo Raggioli, Jennifer Winter

**Affiliations:** 1 Max-Planck Institute of Immunobiology and Epigenetics, Freiburg, Germany; 2 Institute of Human Genetics, University Medical Centre of the Johannes Gutenberg University, Mainz, Germany; 3 University of Freiburg Faculty of Biology, Freiburg, Germany; Georgia Regents University, United States of America

## Abstract

MicroRNAs of the miR-302 cluster are involved in early embryonic development and somatic cell reprogramming. Expression of the miR-302 gene is regulated by the binding of the pluripotency factors Oct4, Sox2 and Nanog to the miR-302 promoter. The specific expression pattern of the miR-302 gene suggested that additional transcription factors might be involved in its regulation. Here, we show that the miR-302 promoter is a direct target of the Wnt/β-catenin signaling pathway. We found that the miR-302 promoter contains three different functional Tcf/Lef binding sites. Two of the three sites were located within the cluster of Oct4/Sox2/Nanog binding sites and were essential for Wnt/β-catenin-mediated regulation of the miR-302 gene. Tcf3, the only Tcf/Lef factor that bound to the miR-302 promoter, acted as a repressor of miR-302 transcription. Interestingly, mutations in the two Tcf/Lef binding sites and the Oct4/Nanog binding sites abolished miR-302 promoter responsiveness to Wnt signaling, suggesting that the Tcf/Lef and the Oct4/Nanog sites interact genetically.

## Introduction

The miR-302 gene encodes a cluster of five microRNAs (miRNAs) (miR-302a/b/c/d and miR-367) that are highly expressed in human embryonic stem cells (ESCs), induced pluripotent stem cells (iPSCs), and mouse embryonal carcinoma cells (e.g., P19 cells) and moderately expressed in mouse ESCs [1–3]. In ESCs and P19 cells, these miRNAs are rapidly downregulated after the induction of differentiation. In the mouse embryo, expression of the miR-302 cluster commences at embryonic day 6.5 (E6.5), peaks at E7.5 and decreases at E8.5 [4,5]. MiRNAs of the miR-302 cluster have important functions in ESC biology and during reprogramming of somatic cells into iPSCs [6–11]. In *Xenopus laevis* embryos, the miR-302 ortholog miR-427 promotes mesendoderm formation by targeting the Lefties, inhibitors of the Nodal pathway [5].

Members of the miR-302 and miR-290 clusters share the same seed sequence, and therefore, it has been proposed that they also share targets and act redundantly. ESCs proliferate rapidly by a mechanism thought to be mediated by a shortened G1 phase. *Dgcr8*-knockout ESCs, which lack all miRNAs, were reported to have a proliferation defect caused by an accumulation of cells in the G1 phase of the cell cycle. Reintroducing miRNAs from the miR-302 or miR-290 clusters rescued the proliferation defect in these cells. Furthermore, these ESC-specific cell cycle-regulating (ESCC) miRNAs were shown to directly target several inhibitors of the cyclin E-CDK2 regulatory pathway, including p21, Rbl2 and Lats2 [12].

MiR-302 cluster miRNAs play important roles during the reprogramming of mouse and human somatic cells to pluripotency. Thus, expression of miR-302 cluster miRNAs rapidly and efficiently reprogram mouse and human fibroblasts to an iPS state without the need for exogenous transcription factors [8].

The polycistronic cluster of miRNAs 302-367 is located within the first intron of a noncoding transcript consisting of three exons and two introns. The promoter region of the miR-302 gene has been characterized in some detail. Thus, it has been shown that the pluripotency factors Oct4, Sox2 and Nanog bind to the promoter region and drive the expression of the miR-302 cluster in human ESCs and in P19 cells [4,13]. Moreover, it has been suggested that the miR-302 gene might be a direct target of EOMES during the specification of the definitive endoderm [14].

Wnt/β-catenin signaling is essential for multiple developmental processes. In the mouse embryo, the Wnt/β-catenin pathway may not be active at pre-implantation stages. At E6.0, before emergence of the primitive streak, Wnt/β-catenin signaling is activated and responsible for primitive streak development and mesendoderm formation [15–22]. The relevance of the Wnt/β-catenin pathway to ESC maintenance remains controversial. Some studies have shown that activation of the Wnt/β-catenin pathway supports pluripotency, whereas other studies indicate a role in promoting differentiation [23]. Recently, mechanisms of TCF-independent β-catenin effects on mouse ESCs were identified by a study showing that β-catenin can form complexes with Oct4 and Klf4 [24,25].

Oct4, Sox2 and Nanog are essential factors that bind to the miR-302 promoter and activate its transcription. The miR-302 cluster is only expressed at moderate levels in mouse ESCs and shows a restricted expression pattern in the mouse embryo with exclusion from preimplantation stages; therefore, the regulation of miR-302 cluster miRNA expression is more complex than has been previously thought. Here, we show that the Wnt/β-catenin pathway promotes transcription of the miR-302 cluster in mouse ESCs and P19 cells.

## Materials and Methods

### Constructs, in vitro mutagenesis

SuperTOPflash, SuperFOPflash and pGL3B-Axin2 (containing the promoter of Axin2) constructs were described previously [26,27]. The miR-302 promoter fragment (positions -968 to +4) was cloned into the multiple cloning site of the pGL4.10 firefly luciferase reporter vector using the restriction enzymes XhoI and BglII. To introduce mutations into this construct, site-directed mutagenesis was performed using Phusion High-Fidelity DNA Polymerase (Finnzymes). A standard cycling reaction (50 µl) containing the original plasmid (50 ng), 10 µl GC buffer, 0.5 µl dNTPs (10 mM; Fermentas), 0.5 µM of each mutagenesis primer containing the desired mutation and 1 U Phusion polymerase was conducted. The following conditions were applied: initial denaturation at 98°C for 1 min; 21 cycles of denaturation (98°C, 10 s), hybridization (58°C, 30 s), and elongation (72°C, 1 min per kbp); final elongation at 72°C for 10 min.

Following temperature cycling, *Dpn*I (New England BioLabs) was added, and the mixture was incubated for 2 hours at 37°C. Five microliters of the mixture were used to transform 50 µl of chemically competent cells.

### Cell culture

Mouse ESCs [28,29] were cultured in DMEM supplemented with 15% FCS, penicillin, streptomycin, glutamine, non-essential amino acids, sodium pyruvate, β-mercaptoethanol and LIF (in-house produced). P19 cells [30] were cultured in DMEM supplemented with 10% FBS, penicillin, streptomycin, glutamine and non-essential amino acid. Mouse ESCs were maintained on a layer of irradiated mouse embryonic fibroblasts. At the beginning of each experiment, cells were transferred to gelatin-coated cell culture dishes. P19 cells were cultivated on gelatin-coated dishes. Cells were treated with 100 ng/ml Wnt3a (PeproTech) or Dkk1 (R&D Systems) the day after seeding. siRNAs were purchased from Qiagen (β-catenin, si β-cat 1), Life Technologies (β-catenin, si β-cat 2; Tcf3) and Dharmacon (control). Transfections using Lipofectamine 2000 (Invitrogen) were carried out according to the manufacturer’s instructions. For genetic ablation of β-catenin, the SR1-Cre-ERT2 mESCs (β-cat^flox/-^: CreER^T2^) were treated with 4-hydroxy-tamoxifen (50 ng/ml; Sigma Aldrich) for 3 days. siRNA sequences are given in Table S1 in File S1.

### Quantitative RT-PCR

Total RNA from mouse ESCs and P19 cells was extracted using the RNeasy Plus Mini Kit (Qiagen) and reverse transcribed using the RevertAid First Strand cDNA Synthesis Kit (Fermentas, Sankt Leon Rot, Germany). Quantitative PCR was performed using the ABsolute QPCR ROX Mix (Thermo Scientific) in combination with the mouse Universal Probe Library (Roche) according to the manufacturer’s instructions. For qPCR of the pri-miR-302 transcript, Absolute QPCR SYBR Green ROX Mix (Thermo Scientific) was used. Primer sequences and universal probe numbers are listed in Table S2 in File S1. MiRNAs were purified from the cell lines using the mirPremier microRNA Isolation Kit (Sigma-Aldrich) according to the manufacturer’s instructions. Mature miRNAs were reverse transcribed with miRNA-specific primers provided by TaqMan MicroRNA Assays using the TaqMan MicroRNA Reverse Transcription Kit. Ten nanograms of RNA was reverse transcribed to cDNA for each specific miRNA. Following reverse transcription of specific miRNAs, qRT-PCR was carried out using TaqMan Fast Universal PCR Master Mix (2x) (Life Technologies) and reagents provided by TaqMan MicroRNA Assays.

### Immunoblotting and ChIP experiments

Cells were lysed by the addition of Magic-Mix [48% urea, 15 mM Tris-HCl (pH 7.5), 8.7% glycerin, 1% SDS, 0.004% bromophenol blue, 143 mM mercaptoethanol], centrifuged through Qia Shredder Columns (Qiagen, Hilden, Germany) and boiled for 5 min at 95°C. Thirty micrograms of total protein from each sample was resolved on 10% SDS-Page gels and blotted onto PVDF membranes. The antibodies used in this study were anti-β-catenin (BD Transduction), anti-GAPDH (Calbiochem) and anti-Oct-3/4 (Santa Cruz; detecting both Oct-3/4 variants).

ChIP experiments were performed as described previously [24]. In brief, 3-4x10^6^ cells (P19 or ESCs) were seeded in 15 cm dishes coated with gelatin one day prior to starting the procedure. Cells were washed once with PBS and subsequently fixed by addition of 1% formaldehyde solution in PBS. Plates were placed on a rocking platform at room temperature for 10 min. After rinsing twice with ice-cold PBS, cells were scraped off and collected by centrifugation (1400 rpm, 5 min, 4°C). The pellet was lysed by adding 500 µl of lysis buffer (containing a mix of protease inhibitors) per 15-cm dish and incubating on ice for 15 min. Sonication with the Covaris S2 sonication system was performed to promote chromatin fragmentation. The lysate was cleared from cellular debris by centrifugation (14000 rpm, 10 min, 4°C). DNA concentration was measured using a NanoDrop 1000 spectrophotometer. For chromatin immunoprecipitation, 250 µg of chromatin was diluted with dilution buffer (20 mM Tris/HCl pH 8.0; 1% Triton X-100; 150 mM NaCl; 2 mM EDTA, containing a mix of protease inhibitors) to a final volume of 1 ml. An aliquot of the chromatin sample (input control) was removed and stored at -20°C. After pre-clearing with protein G dynabeads (Invitrogen), 5 µg of the respective antibody was added, and the samples were incubated on a rotating wheel over night at 4°C. The following day, an equal volume of protein G dynabeads was added to each sample and incubated on a rotating wheel for 1 hour at 4°C. Beads were collected using a magnetic rack and subjected to sequential washings: 2x low salt wash buffer, 2x high salt wash buffer, 1x LiCl wash buffer and 3x TE buffer. Each washing step was performed at 4°C on a rotating wheel for 10 min. Antibody-protein complexes were eluted by adding 100 µl of freshly prepared elution buffer to the beads and constantly shaking at 1250 rpm at room temperature for 1 hour. Eluates as well as the input controls were subjected to reverse crosslinking by incubation at 65°C with constant shaking (950 rpm) over night. DNA was purified using the MinElute PCR Purification Kit (Qiagen). DNA was stored at -20°C until used for qPCR analysis.

The antibodies used for the ChIP experiments were anti-Tcf1 (Santa Cruz), anti-Tcf3 (Santa Cruz), anti-Tcf4 (Santa Cruz) or anti-Lef1 (Santa Cruz). Primer sequences are given in Table S3 in File S1.

### Luciferase assays

For the luciferase assays, transfected cells were incubated with NP40 lysis buffer (250 mM KCl; 50 mM Tris/PO_4_ pH 7.8; 10% glycerol; 0,1% NP-40) on a rocking platform for 30 min at 4°C. The lysates were collected and cleared from cellular debris by centrifugation (14.000 rpm, 10 min, 4°C) immediately or prior to measurement. The lysates were stored at -20°C until use. Firefly and *Renilla* luciferase activities were separately measured in duplicate in 96-well microtiter plates using a Centro LB 960 luminometer (Berthold Technologies). The values obtained for the firefly luciferase activities were normalized to the corresponding *Renilla* luciferase activities.

## Results

### β-catenin regulates expression of the miR-302 cluster in mouse ESCs and P19 cells

**Figure 1:**
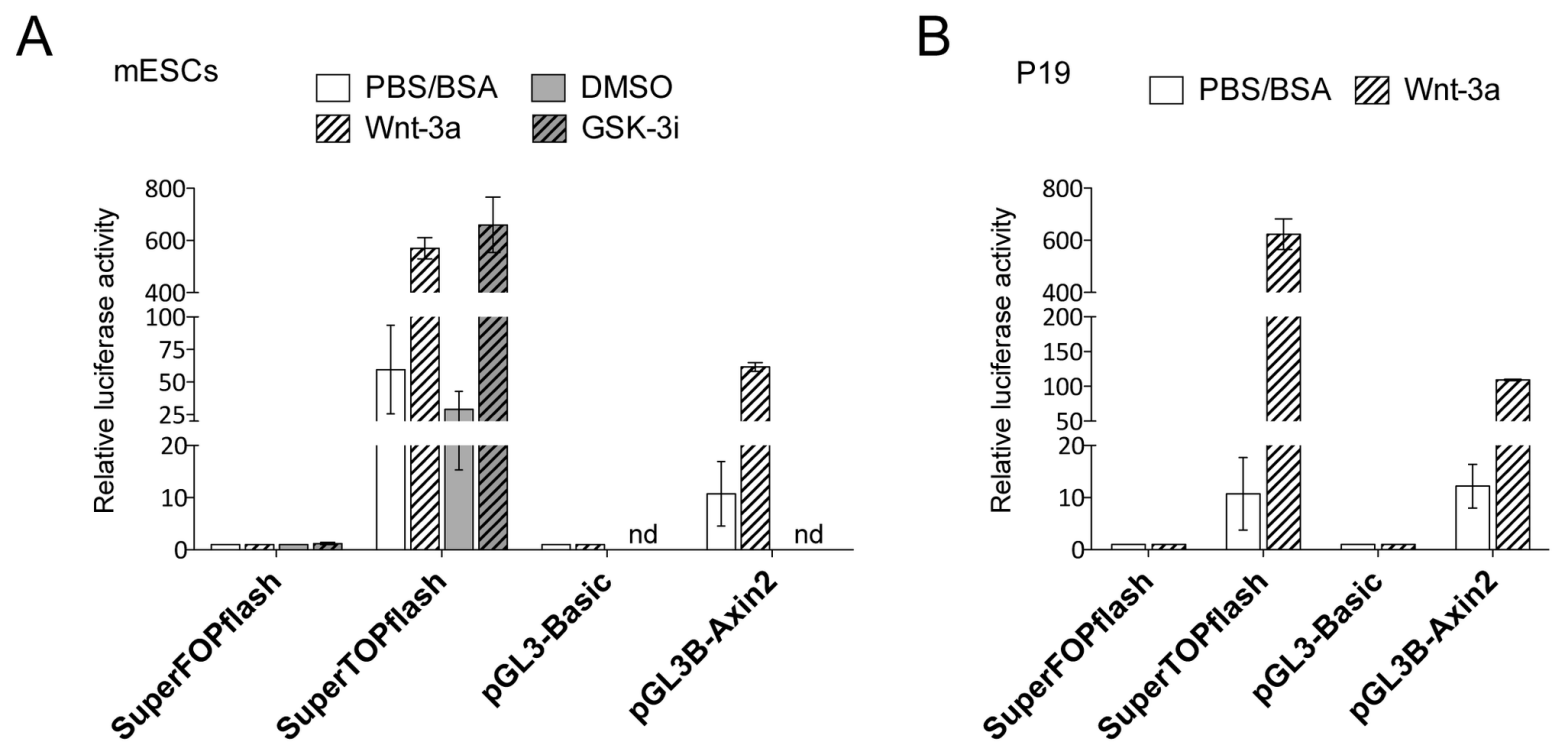
Active Wnt signaling in mESCs and P19 cells. Wnt/β-catenin responsive reporters (SuperTOPflash and pGL3B-Axin2) and their respective corresponding controls (SuperFOPflash and pGL3 Basic) were transfected into (A) mESCs or (B) P19 cells. Cells were either treated with Wnt-3a (Peprotech; 100 ng/ml) or GSK-3 inhibitor (GSK-3i; 10 µM). Luciferase activity was normalized to the respective control plasmid and solvent (set to 1). Data represent the average of three (wt ESCs) or two (P19) replicates ± standard deviation (SD) nd: not determined.

**Figure 2:**
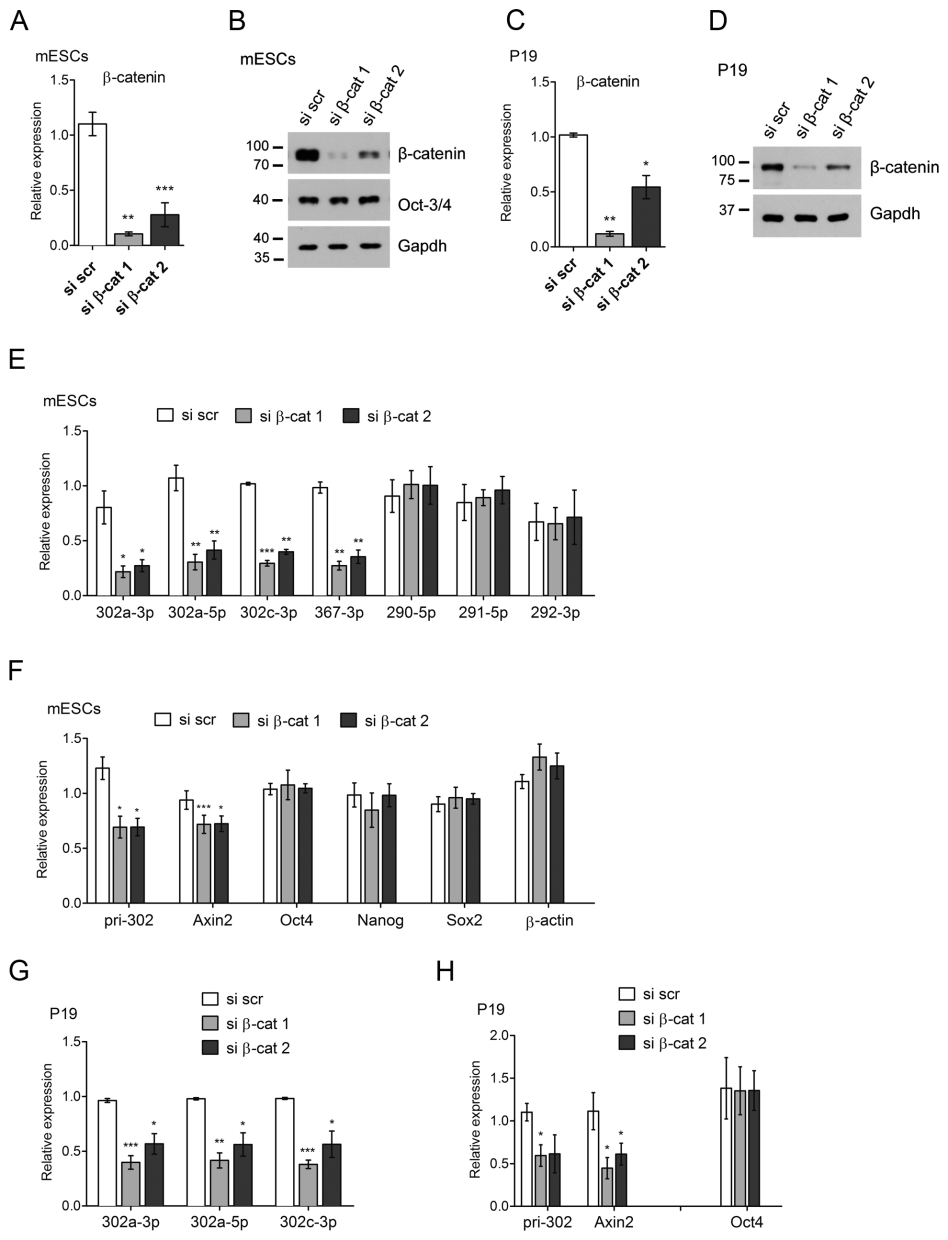
MiR-302 cluster miRNAs are downregulated in β-catenin-depleted cells. β-catenin-specific siRNAs were transfected into mESCs and P19 cells. Fourty-eight hours after the transfection, total RNA, miRNAs or protein was isolated, and cDNA-synthesis and Real Time PCR or Western blot analysis were performed. A–D, efficiency of β-catenin knockdown was determined by qRT-PCR experiments and Western blot analysis in mESCs (A,B) and P19 cells (C,D). E,G, expression levels of mature miRNAs were analyzed by qRT-PCR experiments in wt ESCs (E) and P19 cells (G). F,H, expression levels of the primary *miR-302* transcript (pri-302), the β-catenin target gene *Axin2* and pluripotency-associated genes were quantified using qRT-PCR experiments. *Actb* served as a negative control. Data are depicted as relative expression compared to si scramble control (si scr) (set to 1). Error bars represent SEM of 3-4 independent experiments. Statistical significance over si scr is shown; Student’s t test *p<0.05, **p<0.01, ***p<0.001.

The expression of the miR-302 and miR-290 cluster miRNAs (also known as ESCC miRNAs) correlated with active β-catenin in ESCs, embryonic carcinoma cells and the developing mouse embryo (Figure 1A,B [4,15]). To determine whether β-catenin regulates the expression of the two miRNA clusters, we knocked down β-catenin in mouse ESCs (Figure 2A,B) and analyzed the expression of the mature miR-302 and miR-290 cluster miRNAs by real time RT-PCR. Interestingly, upon knockdown of β-catenin, the expression of all tested miR-302 cluster miRNAs dramatically decreased, whereas the expression of the miR-290 cluster miRNAs did not change, suggesting that β-catenin specifically regulates the expression of the miR-302-cluster miRNAs (Figure 2E). The mature miRNAs of the miR-302 cluster are expressed as a polycistronic primary transcript. To determine whether β-catenin regulates the expression of the primary miR-302 transcript, we carried out real time RT-PCR experiments with primers detecting specifically the primary miR-302 transcript. Indeed, we found that the expression of the primary miR-302 transcript was significantly reduced in the β-catenin knockdown cells compared to cells transfected with a nonsilencing siRNA (Figure 2F). It has been shown previously that the expression of the pri-miR-302 transcript is regulated by the binding of Oct4, Sox2 and Nanog to specific binding sites located in the promoter region of the miR-302 gene. Because β-catenin could potentially regulate the expression of the miR-302 cluster indirectly by regulating the expression of Oct4, Sox2 or Nanog, we also tested for the expression of these transcription factors in the β-catenin knockdown ESCs. The expression of the three transcription factors did not change upon β-catenin knockdown, with the exception of Nanog, which exhibited minimally reduced expression in ESCs transfected with si β-cat 1, but not si β-cat 2 (Figure 2F). This result indicates that the decrease in miR-302 expression observed upon the knockdown of β-catenin is not caused by changes in the expression levels of these transcription factors. However, a decrease in the expression of Nanog (although not significant) as seen in ESCs transfected with si β-cat 1 may further reduce miR-302 expression.

To rule out a cell line-specific effect, we also knocked down β-catenin in the embryonic carcinoma cell line P19 (Figure 2C,D). This cell line also showed reduced expression of both the mature miR-302 miRNAs and the pri-miR-302 transcript upon β-catenin knockdown (Figure 2G, H).

### Expression of the miR-302 cluster is regulated by the Wnt pathway

**Figure 3:**
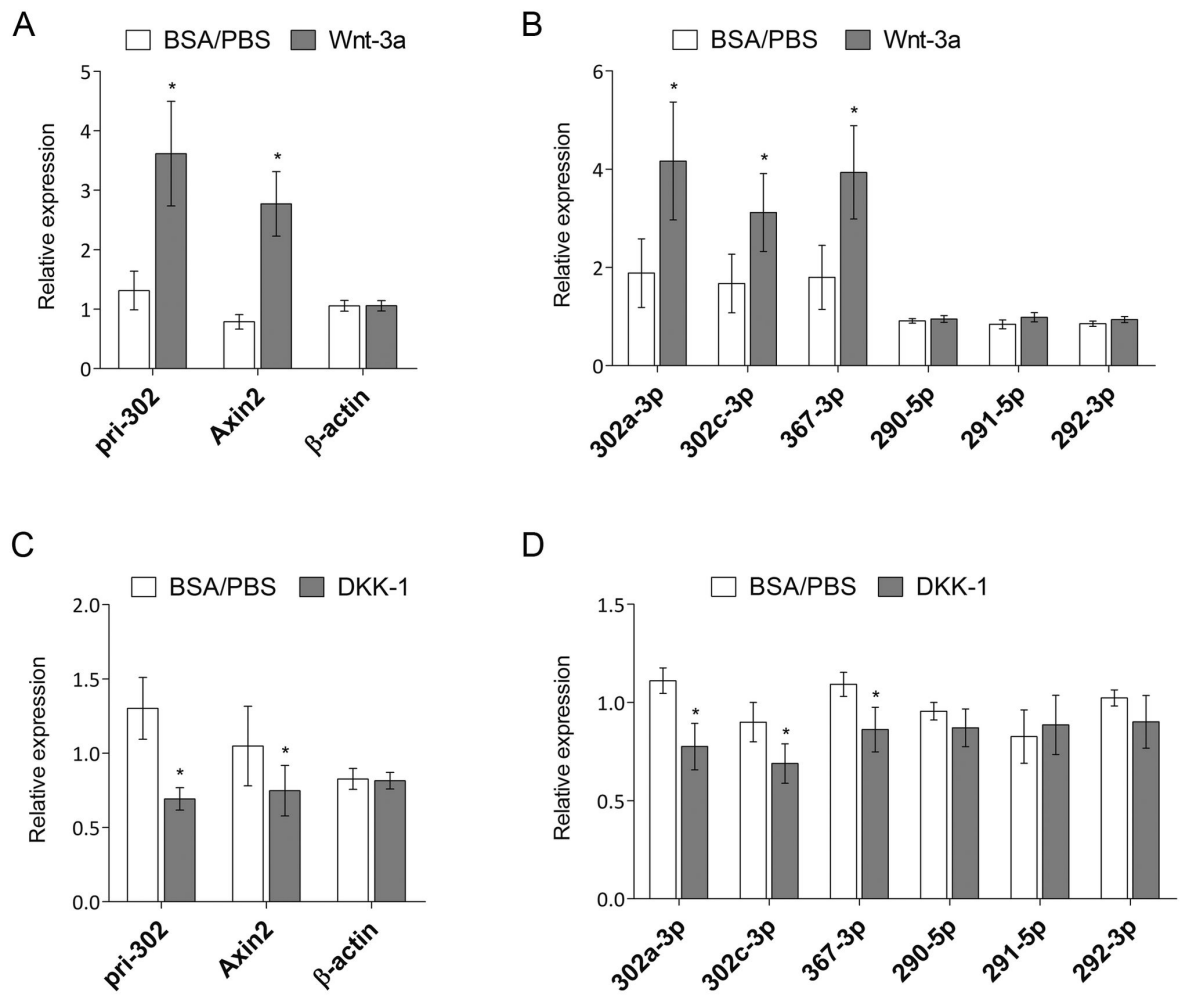
Wnt signaling regulates expression of the miR-302 cluster. A–D, mESCs were incubated with murine Wnt3a (A, B; R&D Systems; 150 ng/ml) or Dkk-1 (C-E; R&D Systems; 100 ng/µl) for 8 hours prior to harvesting and total RNA (A,C) or miRNA (B,D) isolation. A,C, qRT-PCR analysis was performed to determine the expression levels of the primary *miR-302* transcript (pri-302), *Axin2* and *Actb*. B,D: expression levels of mature miRNAs were analyzed by qRT-PCR. Data represent mean values ± SEM from 4–5 independent experiments. Statistical significance over solvent control (BSA/PBS) is shown; Student’s t test *p<0.05, **p<0.01, ***p<0.001.

To determine whether the expression of the miR-302 gene is regulated by Wnt signaling, we stimulated the Wnt pathway by treating mouse ESCs with Wnt3a. Upon treatment, expression of both the pri-miR-302 transcript and the mature miRNAs increased up to three-fold (Figure 3A, B). In contrast, the expression of miRNAs of the miR-290 cluster did not change, indicating that Wnt signaling specifically activated expression of the miR-302 gene (Figure 3B). To determine whether expression of the miR-302 cluster miRNAs is driven by intrinsic Wnt signaling in ESCs, we treated the cells with Dkk1, an inhibitor of the Wnt signaling pathway. The expression of both the primary transcript and the mature miRNAs of the miR-302 cluster, but not the miRNAs of the miR-290 cluster, were significantly downregulated when compared to the control cells (Figure 3C,D). In summary, these results indicate that the expression of the miR-302 gene is under the control of the Wnt signaling pathway in mouse ESCs.

### miR-302 cluster miRNA expression is reduced in inducible β-catenin knockout ESCs

**Figure 4:**
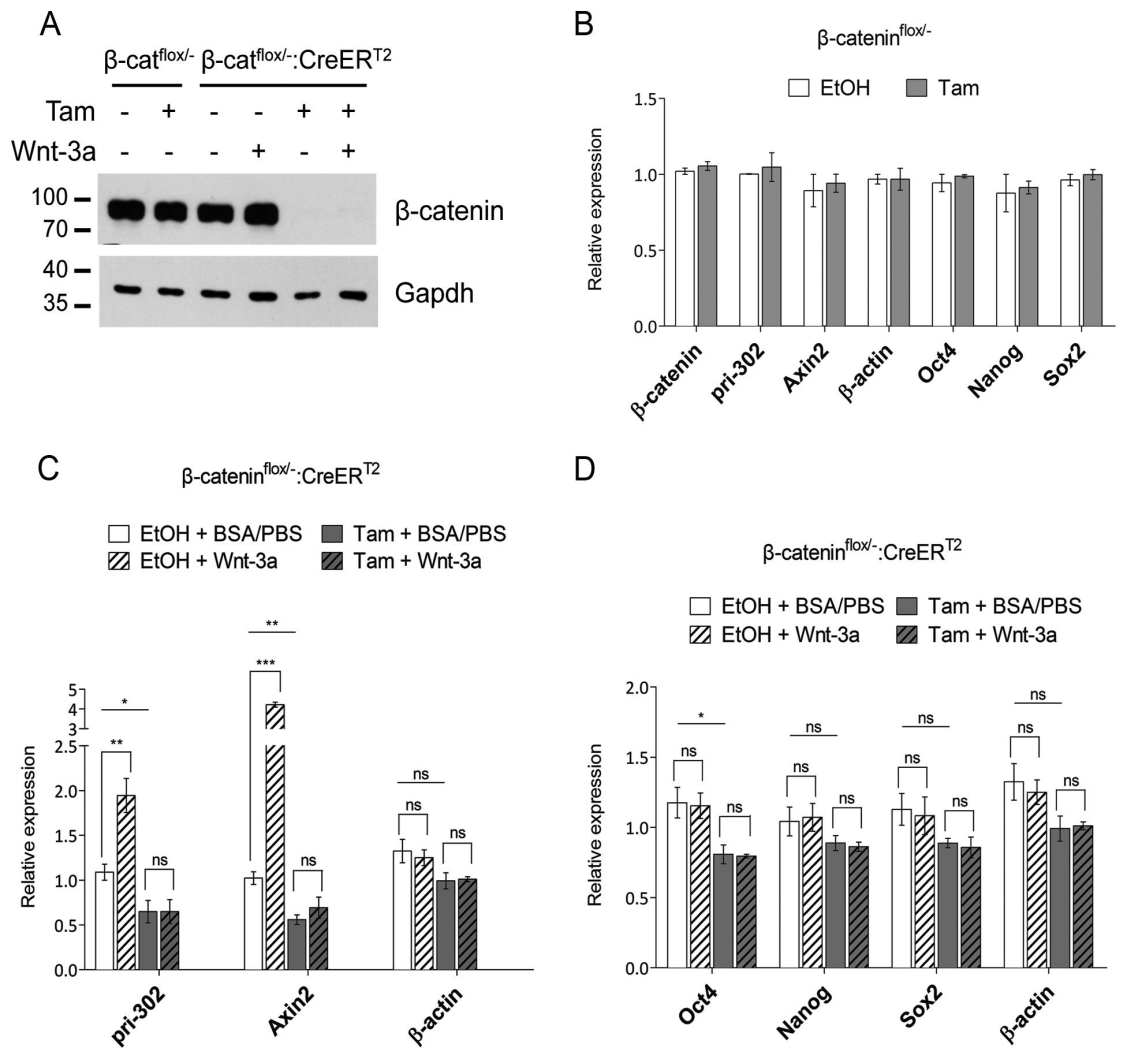
miR-302 cluster miRNAs are downregulated in inducible β-catenin knockout ESCs. A–D, β-catenin^*flox/-*^ or β-catenin^*flox/-*^: *CreER* ^*T2*^ ESCs were treated with 4-hydroxy-tamoxifen (Tam) or control EtOH for 3 days. A, protein levels of β-catenin were determined by Western blot analysis. B, Tam treatment of the parental cell line did not alter gene expression as revealed by qRT-PCR experiments. Bars represent the mean ± SEMof two independent experiments. C, D, Four hours before harvesting, Wnt3a (PeproTech; 100 ng/ml) or control BSA/PBS was administered to β-catenin^*flox/-*^: *CreER* ^*T2*^ ESCs. Gene expression was analyzed using qRT-PCR experiments, and results were normalized to EtOH + BSA/PBS treated cells (set to 1). Data represent the mean of four independent experiments ± SEM C, statistically significant changes in *pri-302* and *Axin2* gene expression upon Wnt-3a or Tam treatment are indicated by asterisks. D, expression of pluripotency-associated genes was analyzed. No statistically significant changes in gene expression could be observed upon Wnt3a treatment. Asterisks above the bars indicate statistical significance of Tam + BSA/PBS treated cells compared to EtOH + BSA/PBS treated cells; Student’s t test *p<0.05, **p<0.01, ***p<0.001, ns: not significant.

To further verify the results of the β-catenin knockdown experiments, we employed tamoxifen-inducible β-catenin knockout ESC lines. Two ESC lines previously established in our department (Raggioli et al., manuscript in preparation) were used. The β-catenin^*flox/-*^: *CreER* ^*T2*^ ESCs were derived from the β-catenin^*flox/-*^ ESCs. The β-catenin^*flox/-*^: *CreER* ^*T2*^ ESCs constitutively express fusion proteins composed of Cre recombinase fused to the ER^T2^ mutant ligand-binding domain of the human estrogen receptor. The recombinase activity of these fusion proteins is blocked by their binding to heat shock proteins. The synthetic ligand 4-hydroxy-tamoxifen (Tam) releases the heat shock proteins and induces recombinase activity [31]. Treatment of the β-cat^flox/-^: CreER^T2^ ESCs with Tam for 3 days led to an efficient depletion of β-catenin from these cells, as evidenced by Western blot analysis (Figure 4A). As a control, treatment of the β-catenin^*flox/-*^ ESCs with Tam, as expected, did not change the level of β-catenin protein nor the expression of the known β-catenin target *Axin 2*; *β-actin*; the pluripotency factor mRNAs *Oct4*, *Sox2* or *Nanog*; or the miR-302 transcript (Figure 4B). In agreement with our results from the knockdown experiments, miR-302 gene expression was significantly reduced in the ESCs genetically ablated for β-catenin (Figure 4C). Furthermore, expression of the miR-302 gene was further upregulated upon Wnt3a treatment in β-cat^flox/-^: CreER^T2^ control cells treated with EtOH, but not in β-cat^flox/-^: CreER^T2^ ESCs treated with Tam (Figure 4C). Expression levels of Oct4 and Sox2 were minimally reduced under these conditions (Figure 4D). These results further indicate that β-catenin regulates the expression of the miR-302 gene as the downstream effector of the Wnt pathway.

### Three Tcf/Lef binding sites regulate the activity of the miR-302 promoter

**Figure 5:**
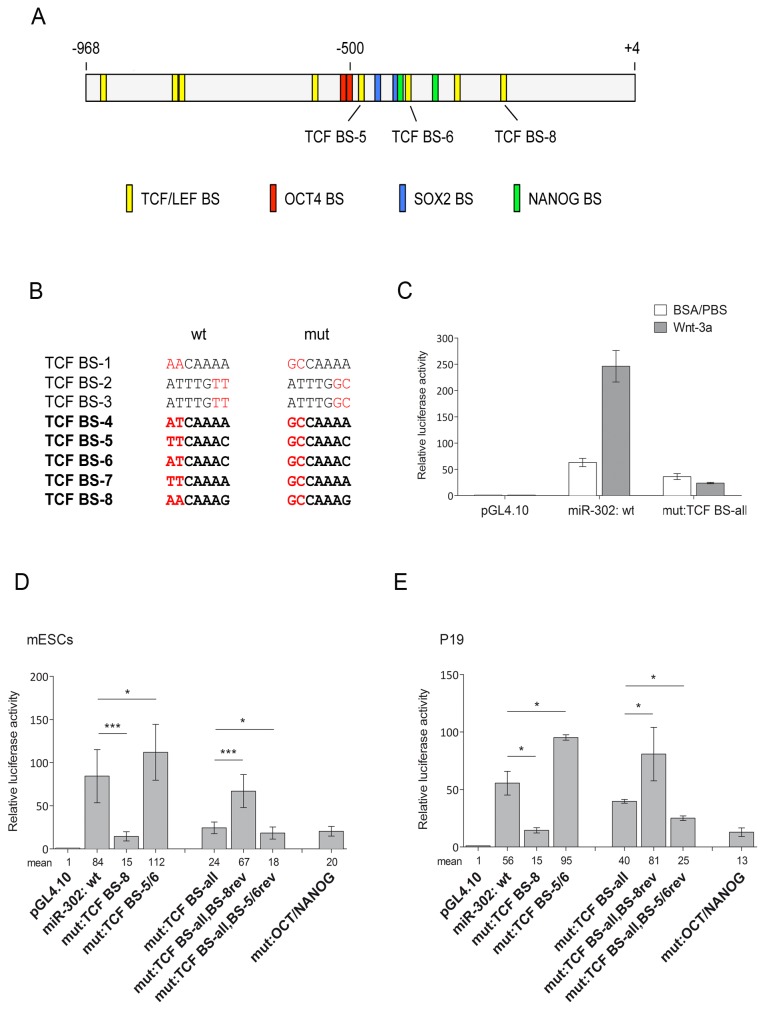
Three Tcf/Lef binding sites regulate the activity of the miR-302 promoter. A, schematic representation of the *miR-302* promoter cloned into the pGL4.10 luciferase vector. Numbers indicate the nucleotide position relative to the first base of the miR-302b stem-loop. Putative Tcf/Lef binding sites (BS) and known BS for Oct4, Nanog and Sox2 are shown by colored boxes. B, candidate Tcf/Lef BS were consecutively numbered starting from the most upstream position. Wildtype (wt) and mutated (mut) sequences are shown. Tcf BS highlighted in bold are conserved between human and mouse. C–E, luciferase assays were carried out in mESCs (C,D) or P19 cells (E) to examine the activity of the miR-302 promoter fragment with or without mutations in all eight candidate Tcf BS (C) or with mutations in BS 8 alone or BS 5 and BS 6 in combination (D,E) upon Wnt-3a treatment (R&D Systems; 150 ng/ml). Data were normalized to the empty vector (pGL4.10) for each treatment (set to 1). Bars represent the mean of at least three independent experiments ± SD with the exception of P19 cells (E), which represent two independent experiments ± SD; Student’s t test *p<0.05, ***p<0.001.

A fragment of the miR-302 promoter containing the binding sites for OCT4, SOX2 and NANOG is sufficient to drive OCT4-dependent activation of the miR-302-367 cluster in human ESCs and P19 cells [4]. To determine whether β-catenin directly regulates the expression of the miR-302 gene, we screened this promoter fragment for potential Tcf/Lef binding sites. In total, we identified eight candidate sites, five of which were conserved between human and mouse (Figure 5A, B). We cloned the miR-302 promoter fragment containing these sites into the pGL4.10 luciferase reporter vector and carried out luciferase experiments. Luciferase activity was minimal after transfection of the control pGL4.10 vector, whereas it increased strongly after transfection of the pGL4.10 vector containing the miR-302 promoter sequence (Figure 5C). Of note, luciferase activity was further enhanced upon treatment with Wnt-3a, indicating that all promoter elements necessary for Wnt/β-catenin-mediated regulation of expression were present in the cloned promoter fragment (Figure 5C). Upon mutation of all eight candidate Tcf/Lef sites, luciferase activity was reduced and was not responsive to Wnt3a treatment, indicating that one or several of the candidate Tcf/Lef sites in the promoter fragment were necessary for Wnt/β-catenin mediated regulation of expression (Figure 5C). Moreover, we performed luciferase assays after mutating individual Tcf/Lef candidate sites and included a construct containing mutations in the Oct4/Nanog binding sites. These assays revealed that three out of the eight candidate sites were important regulators of miR-302 expression. Mutation of the conserved binding site 8 (bs 8), located most proximal to the transcription start site (Figure 5A), significantly reduced luciferase activity (Figure 5D, E). Reversion of only bs 8 to the wild-type sequence in the construct carrying mutations in all candidate Tcf/Lef sites restored luciferase activity. Unexpectedly, the simultaneous mutation of bs 5 and bs 6 slightly increased luciferase activity, whereas their reversion to the wild type sequence in the construct carrying mutations in all eight candidate Tcf/Lef sites further decreased luciferase activity, suggesting that bs 5 together with bs 6 fulfilled a repressive function (Figure 5D,E). These non-canonical Tcf/Lef binding sites, which differed from the consensus sequence T/A-T/A-C-A-A-A-G by the change of G to C at the last position, were located within a cluster of binding sites for the pluripotency factors Oct4, Sox2 and Nanog in the miR-302 promoter (Figure 5A). Both sites were conserved in humans.

### The miR-302 promoter contains non-canonical Tcf/Lef sites that drive expression of the miR-302 cluster miRNAs in a Wnt/β-catenin-dependent manner

**Figure 6:**
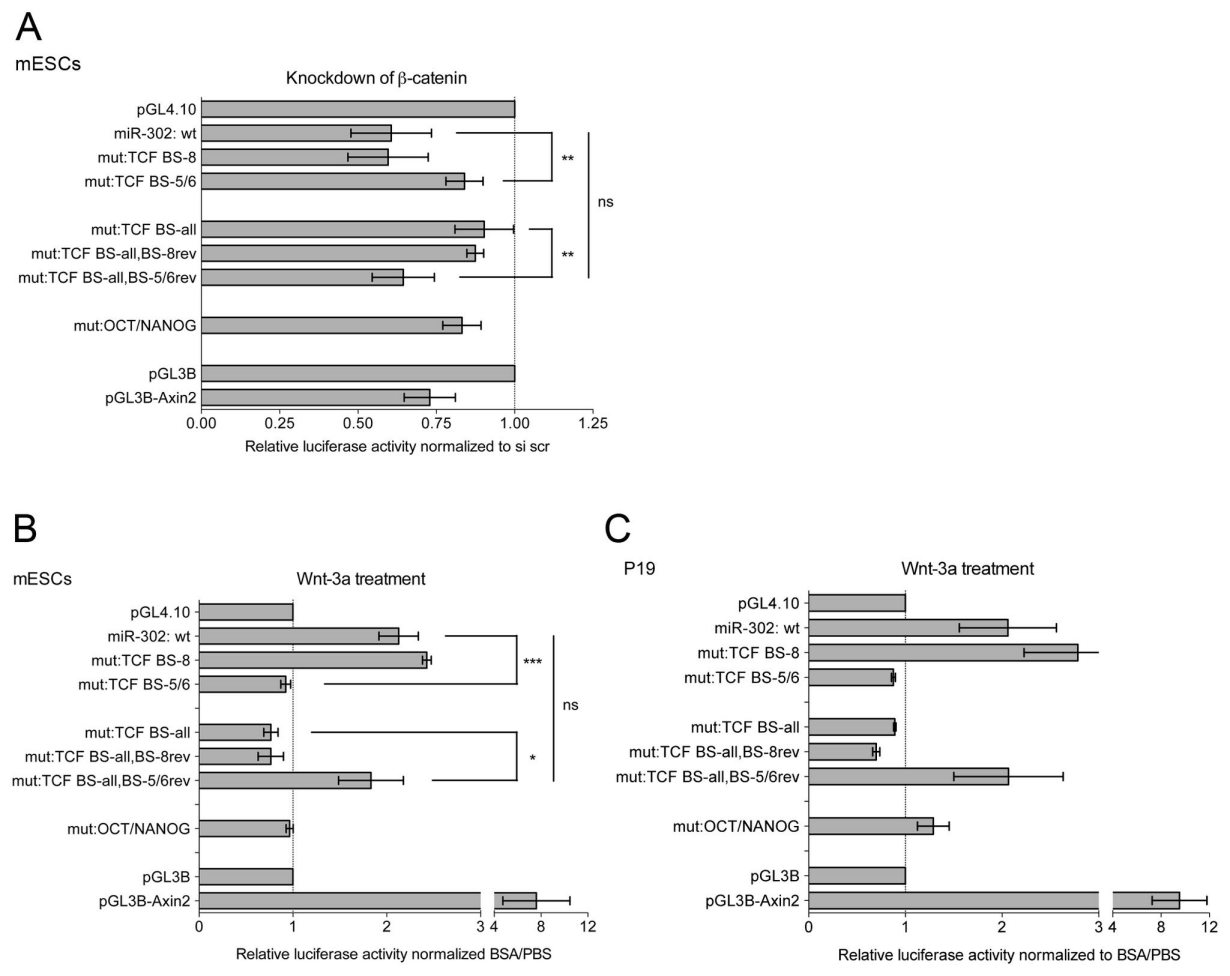
The responsiveness of the *miR-302* promoter to Wnt/β-catenin signaling depends on Tcf BS 5 and Tcf BS 6 and the Oct4/Nanog BS. Luciferase assays were carried out to examine the activities of different pGL4.10-miR-302 promoter constructs upon β-catenin knockdown (A) or Wnt3a treatment (B,C) (Peprotech; 100 ng/ml). Luciferase activity was normalized to the solvent control (BSA/PBS) for each construct. A, mESCs were transfected with β-catenin specific siRNAs. Twenty-four hours after the transfection of siRNAs, the indicated plasmids were transfected and luciferase assays were performed 24 hours later. B,C, mESCS (B) or P19 cells (C) were transfected with the indicated plasmids and treated with Wnt3a. Twenty-four hours later, luciferase assays were performed. Results of three (A,B) or two (C) independent experiments are shown. Error bars indicate standard error of the mean (SEM). Statistical significance is indicated; Student’s t test *p<0.05, **p<0.01, ***p<0.001, ns: not significant. BS: binding site.

To determine which of the three identified binding sites were the effectors of Wnt/β-catenin signaling, we performed two different sets of experiments. First, we knocked down β-catenin in mESCs. Second, we treated mESCs and P19 cells with Wnt3a prior to performing luciferase assays. In agreement with our previous results, β-catenin knockdown repressed the wildtype miR-302 promoter, whereas Wnt3a treatment activated this promoter (Figure 6A–C). Moreover, these effects were abrogated when all eight candidate Tcf/Lef sites were mutated. Interestingly, introducing a mutation specifically into bs 8 did not abrogate the responsiveness of the promoter towards the β-catenin knockdown or Wnt3a treatment. In addition, reintroducing the wildtype bs 8 sequence into the miR-302 promoter construct that carried mutations in all eight candidate Tcf/Lef sites did not restore the responsiveness of this miR-302 promoter fragment towards the β-catenin knockdown or the Wnt3a treatment (Figure 6A–C). These results suggest that although bs 8 seemed to be important for the basal activity of the miR-302 promoter, it did not seem to be the effector of Wnt/β-catenin signaling. The simultaneous mutation of bs 5 and bs 6 completely abolished the responsiveness of the miR-302 promoter fragment to β-catenin knockdown or Wnt3a treatment. Reintroduction of the wildtype bs 5 and bs 6 into the promoter construct containing mutations in all eight candidate Tcf/Lef binding sites restored the responsiveness of this miR-302 promoter fragment to β-catenin knockdown and Wnt3a treatment (Figure 6A–C). Interestingly, mutations in the binding sites for Oct4 and Nanog also abolished the responsiveness of the miR-302 promoter fragment to β-catenin knockdown and Wnt3a treatment, suggesting that the Tcf/Lef and Oct4/Nanog binding sites interacted (Figure 6A–C). Altogether, these results indicate that bs 5 and bs 6 are the effectors of Wnt/β-catenin signaling in the miR-302 promoter.

### Tcf3 binds to the miR-302 promoter and represses miR-302 transcription

**Figure 7:**
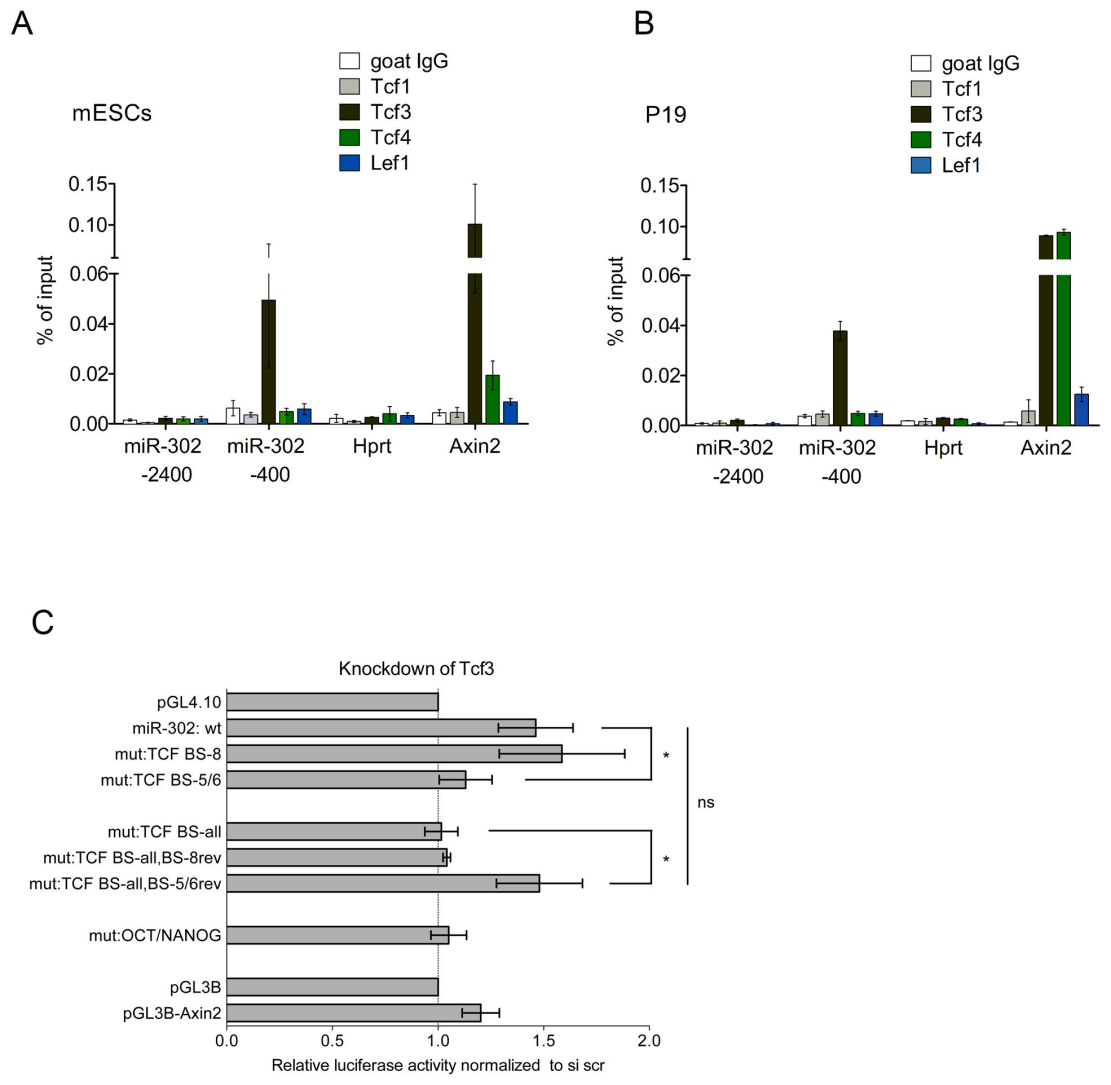
Tcf3 binds to the miR-302 promoter *in* *vivo* and represses its activity. A,B, ChIP experiments were performed in mESCs (A) or P19 cells (B) with antibodies specific for Tcf1, Tcf3, Tcf4 and Lef1. Goat IgG served as a negative control. Precipitated DNA was used to amplify different genomic regions as indicated by qPCR. Data represent the mean of two independent experiments ± SD. C, luciferase assays were performed to examine the activities of different pGL4.10-miR-302 promoter constructs upon Tcf3 knockdown. mESCs were transfected with Tcf3 specific siRNAs. Twenty-four hours after the transfection of siRNAs, the indicated plasmids were transfected, and luciferase assays were performed 24 hours later. Luciferase activity was normalized to the solvent control (BSA/PBS) for each construct. Data represent the mean of four independent experiments ± SD. Statistical significance is indicated; Student’s t test *p<0.05, ns: not significant.

Four Tcf/Lef proteins are expressed in embryonic stem cells: Tcf1, Tcf3, Tcf4 and Lef1. It is thought that Tcf1, Tcf4 and Lef1 fulfill activating functions, whereas Tcf3 mainly acts as a transcriptional repressor. To determine which Tcf/Lef factors bind to the miR-302 promoter, we carried out chromatin immunoprecipitation (ChIP) experiments in mESCs and P19 cells (Figure 7 A, B). We could not find enrichment for any of the activating Tcf/Lef factors on the miR-302 promoter, whereas the repressive factor Tcf3 was enriched at the -400 position of the miR-302 promoter where Tcf/Lef bs 5 and bs 6 were located. To further determine whether Tcf3 represses the transcription of the miR-302 gene, we knocked down Tcf3 in mESCs by transfection of specific siRNAs and carried out luciferase assays (Figure 7C). In agreement with a repressive function of Tcf3, its knockdown increased the luciferase activity of the miR-302 fragment. Mutations in bs 8 did not abolish the activation of the miR-302 promoter fragment upon Tcf3 knockdown, whereas bs 5 and bs 6 mutations simultaneously abrogated the derepressive effect. Reintroduction of the wild type sequences of bs 5 and bs 6, but not bs 8, into the miR-302 promoter construct carrying mutations in all eight candidate Tcf/Lef sites restored the derepressive effect observed upon Tcf3 knockdown. Oct4 and Nanog binding site mutations also abrogated this derepressive effect, suggesting that the Tcf/Lef- and Oct4/Nanog binding sites interact (Figure 7C). In summary, these results show that Tcf3 binds to the miR-302 promoter and represses the transcription of the miR-302 gene through its interaction with the non-canonical Tcf/Lef binding sites 5 and 6.

## Discussion

Here, we identify the miR-302 gene as a new transcriptional target of the Wnt/β-catenin pathway in mESCs and P19 cells. Wnt/β-catenin mediated transcription of the miR-302 gene through two non-canonical Tcf/Lef binding sites bs 5 and bs 6 located in the miR-302 promoter. As the only member of the Tcf/Lef family that demonstrated binding to the miR-302 promoter, Tcf3 bound to the bs 5 and bs 6 positions of the miR-302 promoter and repressed miR-302 transcription through these sites. All other members of the Tcf/Lef family are considered to be transcriptional activators, whereas Tcf3 is a transcriptional repressor [19,32–34]. It was shown that Tcf3 shares an overlapping set of target genes, including the miR-302 gene, with the pluripotency factors Oct4, Sox2 and Nanog in ESCs. However, Tcf3 acts as a repressor on the transcription of these target genes, whereas the pluripotency factors act as activators [35–37]. Our results on the regulation of the miR-302 promoter by Tcf3 further confirm this observation.

It was originally thought that upon activation of the Wnt/β-catenin pathway, β-catenin shuttles from the cytoplasm to the nucleus and activates target gene transcription by replacing members of the family of groucho co-repressors from the Tcf/Lef factors. Subsequent studies revealed that additional mechanisms of action may exist. In addition to its interaction with a number of transcription factors other than the Tcf/Lef factors [24,25,38,39], β-catenin derepresses certain target genes by mediating release of Tcf3 from the target gene’s promoters [23,35,40]. β-catenin might activate the transcription of the miR-302 promoter by a similar derepressive mechanism because, in our study, both the activating effect of β-catenin and Wnt3a as well as the repressive effect of Tcf3 was mediated by the Tcf/Lef binding sites bs 5 and bs 6. Moreover, mutating these binding sites under basal conditions resulted in an increase in luciferase activity, indicating that these sites were bound by a repressor.

Bs 5 and bs 6 are located within a cluster of binding sites for the pluripotency factors Oct4, Sox2 and Nanog in the miR-302 promoter. Because of their close proximity to each other, these binding sites might genetically interact. This idea is supported by the fact that the responsiveness of the miR-302 promoter to β-catenin knockdown and the Wnt3a treatment was abolished not only when Tcf/Lef binding site bs 5 and bs 6 were mutated but also when the Oct4/Nanog binding sites were mutated. Moreover, the derepressive effect observed upon depletion of Tcf3 from mESCs was abrogated not only by the simultaneous mutation of bs 5 and bs 6 but also by the mutation of the Oct4/Nanog sites. An interdependence of the Tcf/Lef sites and the Oct4/Nanog sites is further supported by the specific expression pattern of the miR-302 cluster in the mouse embryo. In mESCs derived from the E3.5 blastocyst, the miR-290 cluster miRNAs are expressed at much higher levels than the miRNAs of the miR-302 cluster [41]. In contrast, miR-302 cluster miRNAs are highly expressed in P19 cells, a cell line originally derived from an E7.5 embryo [1,2,30]. In agreement with their *in vitro* expression pattern, the expression of miR-290 cluster miRNAs is already upregulated in four-cell stage mouse embryos and reaches the highest level in the blastocyst, whereas miRNAs of the miR-302 cluster begin expression later at E6.5 and reach their expression peak at E7.5 [4,42,43]. These cluster-specific expression patterns suggest that miRNAs from the two clusters are important during different stages of embryonic development. The miR-290 cluster miRNAs may have important functions in mESCs and *in vivo* in the blastocyst, whereas miR-302 cluster miRNAs may be important at later stages. Although it was clearly demonstrated that Oct4, Sox2 and Nanog regulate the expression of the miR-302 gene, the expression pattern of these pluripotency factors and the miR-302 gene only partially overlap [4]. Thus, Oct4, Sox2 and Nanog are highly expressed in the blastocyst and beyond until E7.5, whereas the expression of the miR-302 gene is restricted to E6.5 and E7.5. This result suggests that transcription factors other than Oct4, Sox2 and Nanog may be additionally involved in the regulation of miR-302 transcription. In agreement, EOMES has been suggested to directly regulate expression on the miR-302 gene [14]. Although β-catenin is expressed early in mouse development, active Wnt signaling has only been detected in prestreak embryos from E6 onwards [15,16]

The activation of the Wnt pathway perfectly mirrors the increase in miR-302 gene expression, and thus, active Wnt/β-catenin signaling could be another missing factor that drives miR-302 expression in the mouse embryo. This idea is further supported by the fact that the miR-302 cluster functionally overlaps with the Wnt/β-catenin pathway. The miRNAs from the miR-302 cluster, in addition to Wnt/β-catenin signaling, promote the formation of mesendoderm in the gastrulating embryo, whereas the loss of Tcf3 results in the expansion of axial mesoderm in the mouse [5,15,19].

Several of the miR-302 and miR-290 cluster miRNAs share similar seed sequences, and it has been suggested that miRNAs of the two clusters act redundantly [12]. Therefore, it was of general interest to determine whether, in addition to the miR-302 gene, the miR-290 gene was under the transcriptional control of the Wnt/β-catenin pathway. In contrast to the miRNAs of the miR-302 cluster, however, the miRNAs of the miR-290 cluster did not alter their expression upon β-catenin depletion from mESCs or P19 cells. This observation was in contrast to findings in human studies that showed that Wnt/β-catenin signaling regulates the transcription of the miR-371-373 cluster, the human ortholog of the mouse miR-290 cluster [44]. One explanation for this discrepancy might be species-specific differences. A cell type-specific effect, however, cannot be excluded (the Zhou et al. study was carried out in human cancer cells). In contrast, we focused on transcriptional regulation of the miR-290 cluster in mESCs and mouse embryonic carcinoma cells.

## Supporting Information

File S1 Table S1, siRNA sequences. Table S2, Primer sequences and numbers of universal probes used in the qPCR experiments. Table S3, Sequences of the primers used in ChIP experiments.
(DOCX)
