# Supplementary material for: The Wnt/β-Catenin Pathway Regulates the Expression of the miR-302 Cluster in Mouse ESCs and P19 Cells
Source: PLoS One. 2013 Sep 10;8(9):e75315. doi: 10.1371/journal.pone.0075315 (PMC3769259; doi:10.1371/journal.pone.0075315)
Supplement: File S1 — Table S1, siRNA sequences. Table S2, Primer sequences and numbers of universal probes used in the qPCR experiments. Table S3, Sequences of the primers used in ChIP experiments. (DOCX) [file pone.0075315.s001.docx]

Table S1: siRNA oligonucleotides

| **Target gene** | **Distributor** | **Indication in the text** |
| --- | --- | --- |
| *Ctnnb1*  (β-catenin) | HP GenomeWide siRNA  Qiagen, SI00942046 | si β-cat 1 |
| *Ctnnb1*  (β-catenin) | Silencer Select Pre-designed siRNA  Life Technologies, s63418 | si β-cat 2 |
| *Tcf7l1* (Tcf3) | Silencer Select Pre-designed siRNA  Life Technologies, s74836 | si Tcf3 1 |
| *Tcf7l1* (Tcf3) | Silencer Select Pre-designed siRNA  Life Technologies, s74835 | si Tcf3 2 |
| negative control | ON-TARGET*plus* Non-targeting siRNA #1  Dharmacon, D-001810-01-05 | si scr |

Table S2: Primer sequences and probes for qPCR

| **Gene** | **Forward** **(5’ → 3’)** | **Reverse** **(5’ → 3’)** | **Universal probe** |
| --- | --- | --- | --- |
| Axin2 | gagagtgagcggcagagc | cggctgactcgttctcct | 96 |
| β-actin | taaggccaaccgtgaaaaga | accagaggcatacagggaca | 64 |
| β-catenin | tgacacctcccaagtccttt | ttgcatactgcccgtcaat | 48 |
| Nanog | ttcttgcttacaagggtctgc | agaggaagggcgaggaga | 110 |
| Oct-3/4 | gttggagaaggtggaaccaa | ctccttctgcagggctttc | 95 |
| Sox2 | tccaaaaactaatcacaacaatcg | gaagtgcaattgggatgaaaa | 63 |
| Tbp | cggtcgcgtcattttctc | gggttatcttcacacaccatga | 107 |
| pri-miR302 | CTGTGGGTTTGCTCTTCTGTTTT | GAGACAGAAAGCATTCCCATGTT |  |

Table S3: Primer sequences for ChIP experiments

| **Gene/region** | **Forward (5’ → 3’)** | **Reverse (5’ → 3’)** |
| --- | --- | --- |
| Axin2 | GTTAAATCCACAGCGCAGTTT | ttcaacccaggtcctgtttc |
| Hprt | AACGACTTTAGCCTGCTCTATTCT | TCAACCAACAAGTAACCAGATACC |
| miR-302 -2400 | tcaccatatcattctccactgaa | cgaatagcctgtacacctttcttt |
| miR-302 -400 | caaacgggcagataggagat | actgctaaaaccattaccacaaga |
